# Damped Linear Response TDDFT with Range-Separated Functionals and Density Fitting

**DOI:** 10.1021/acs.jpca.5c03798

**Published:** 2025-09-26

**Authors:** Pierpaolo D’Antoni, Daniele Toffoli, Mauro Stener

**Affiliations:** † Dipartimento di Scienze Chimiche e Farmaceutiche, 9315 Università di Trieste, Via Giorgieri 1, 34127 Trieste, Italy; ‡ IOM-CNR, Istituto Officina dei Materiali, Basovizza SS-14, Km 163.5, 34149 Trieste, Italy

## Abstract

The Resolution of the Identity (RI) technique has been employed to enhance the computational efficiency of Range-Separated (RS) exchange–correlation (xc-) functionals at the time-dependent density functional theory (TDDFT) level using the Hybrid Diagonal Approximation. RI has been implemented within the polTDDFT algorithm (a complex damped polarization method) as part of the AMS/ADF program suite. Comparison between the present TDDFT simulations and the Casida algorithm, for both the model ethylene–tetrafluoroethylene charge-transfer system and the experimental photoabsorption spectrum of a large donor–acceptor–acceptor triad, demonstrates the method’s excellent accuracy and efficiency. The present method provides a reliable and computationally efficient tool to predict photoabsorption spectra in the presence of charge-transfer transitions and is suitable for applications to large systems with sizes up to several hundred atoms.

## Graphical abstract

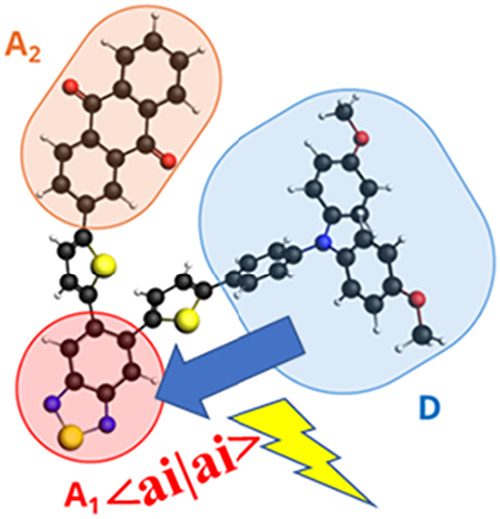

## 1 Introduction

One of the most direct and easily applicable experimental methods to probe the electronic structure of matter, both in general and for specific materials, is optical spectroscopy. Although the information contained in an electronic spectrum is very rich, it is often challenging to extract it without a robust and reliable theoretical model. Such a model enables, for instance, the assignment of experimental features to specific electronic transitions. For this reason, the availability of suitably accurate computational methods to support and rationalize experiments is of great importance for the scientific community.^1^ Moreover, when the computational approach is accurate enough to be predictive, it can be employed for the rational design of new materials with targeted optical properties. This is particularly relevant in fields such as solar energy conversion and light harvesting, where developing materials with an increasingly high efficiency is crucial.

In this context, a reliable theoretical tool for solar cell materials must be able to describe the charge-transfer (CT) excitations, which are fundamental to generate the exciton, which may evolve into charge separation and ultimately lead to photocurrent generation.

From a theoretical point of view, the optimal compromise between accuracy and computational economy is currently offered by time-dependent density functional theory (TDDFT), which allows the calculation of systems containing up to a thousand atoms. In quantum chemistry, the most widely used implementation of TDDFT is that proposed by Casida.^2^ In the Casida formulation, an eigenvalue equation is solved on the space of one-electron excitations, the number of which is the product of the number of occupied and unoccupied orbitals. In addition to Casida’s approach, other TDDFT formulations and implementations are available.^3–11^ More recently, a damped linear response TDDFT algorithm, which extracts the absorption spectrum from the average of the complex polarizability tensor^12–14^ (also known as polTDDFT), has proven particularly suitable for application to very large systems,^15^ and will be employed also in the present work.

In both DFT and TDDFT, accuracy remains a critical open challenge, as it is intimately connected with the choice of the exchange–correlation (xc-) functional: in DFT to solve Kohn–Sham equations, and in the response kernel of TDDFT when solving the TDDFT equations.^16^ The quest to improve the accuracy and efficiency of xc-functionals and kernels is an active and ongoing field of research.^17,18^ Hybrid xc-functionals for DFT and hybrid kernels for TDDFT, i.e., functionals including a variable fraction of the exact Hartree–Fock (HF) nonlocal exchange, often represent the best choice in terms of accuracy, at least with respect to simpler Generalized Gradient Approximation, GGA. Among hybrid xc-functionals, B3LYP^19,20^ is the most popular. However, even hybrid functionals are known to fail in the description of CT excitations. This failure is well understood: since CT excitations involve a pair of occupied-virtual orbitals lying in different spatial regions, the kernel must contain the full nonlocal Fock exchange to recover the particle–hole interaction.^21^

From a computational point of view, for global hybrid and RS kernels, the HF nonlocal exchange is demanding when evaluating the matrix elements needed for the Casida equations. More precisely, matrix elements can be treated efficiently when Gaussian Type Orbitals (GTO) basis sets are employed, but become problematic with other basis sets, such as Slater Type Orbitals (STO) or Plane Waves (PW). A recently proposed approximate scheme, known as the Hybrid Diagonal Approximation^22^ (HDA), addresses this issue by including the nonlocal exchange only in the diagonal terms of the response equations. This reduces the computational cost to that of a conventional GGA kernel, while retaining nearly the same accuracy as full hybrid TDDFT. More recently, an improved HDA implementation exploiting the Resolution of Identity (RI) technique, named fitted HDA, has proven extremely efficient^23^ and gives almost quantitative agreement with respect to high-resolution low-temperature photoabsorption experiments for gold nanoalloys protected by thiol ligands.^24,25^

In the present work, we extended the previous fitted HDA implementation from global hybrid (like B3LYP) to RS functionals using STO basis set within the ADF engine of the AMS program suite.^26^ For the moment, we focus on the RS CAMY-B3LYP functional.^27^ The Review is organized as follows. First, we provide a short review of the HDA approximation in both the Casida and polTDDFT implementations. Then, we detail the RI method to calculate HDA integrals and describe its implementation within the AMS-ADF program. Finally, to assess the performance of the method, we applied it to two systems: the prototypical ethylene–tetrafluoroethylene adduct, often used to benchmark CT transitions, and a realistic donor–acceptor–acceptor triad exhibiting intramolecular CT excitations.

## 2 Theoretical Method and Implementation

The present implementation of the Hybrid Diagonal Approximation (HDA)^22^ is developed within the damped linear response complex polarizability algorithm (called polTDDFT). A brief summary of the HDA scheme is provided below, while a detailed derivation can be found in the literature. The linear response TDDFT equations within the Random Phase Approximation (RPA) take the standard form

1 $$\left(\begin{array}{ll} A & B\\ {\begin{array}{l} B \end{array}}^{*} & A^{*} \end{array}\right)\left(\begin{array}{l} X\\ Y \end{array}\right)=\omega \left(\begin{array}{ll} 1 & 0\\ 0 & -1 \end{array}\right)\left(\begin{array}{l} X\\ Y \end{array}\right)$$

where the submatrices *A* and *B* take the following general form for hybrid kernels

2a $$A_{\mathrm{ia},\mathrm{jb}}=\delta_{\mathrm{ij}}\delta_{\mathrm{ab}}\left(\varepsilon_{a}-\varepsilon_{i}\right)+\left\langle \mathrm{aj}\right|\mathrm{ib}\rangle -\alpha \left\langle \mathrm{aj}\right|\mathrm{bi}\rangle +\left(1-\alpha \right)\left\langle a\left|\frac{\partial V_{\mathrm{XC}}}{\partial \rho }j^{*}b\right|i\right\rangle$$

2b $$B_{\mathrm{ia},\mathrm{jb}}=\left\langle \mathrm{ab}\right|\mathrm{ij}\rangle -\alpha \left\langle \mathrm{ab}\right|\mathrm{ji}\rangle +\left(1-\alpha \right)\left\langle a\left|\frac{\partial V_{\mathrm{XC}}}{\partial \rho }b^{*}j\right|i\right\rangle$$

where in expressions [Disp-formula eq2] and 3, the indices *i*, *j* refer to occupied orbitals and *a, b* refer to the virtual ones. α represents the fraction of nonlocal HF exchange in the xc-kernel and the Adiabatic Local Density Approximation, ALDA, is assumed in the last terms of both equations. The two-electron integrals in the last terms of eqs 2a and 3 correspond to the elements of the coupling matrix with the ALDA local part of the time-independent *f_xc_* kernel, namely

3 $$\left\langle a|\frac{\partial V_{\mathrm{XC}}}{\partial \rho }j^{*}b|i\right\rangle =\iint \varphi_{a}^{*}\left(r_{1}\right)\varphi_{j}^{*}\left(r_{2}\right)f_{\mathrm{xc}}^{ALDA}\left(r_{1},r_{2}\right)\varphi_{i}\left(r_{1}\right)\varphi_{b}\left(r_{2}\right)dr_{1}dr_{2}$$

A general definition of the kernel is given in eq (7.89) from the Ullrich book.^28^

In practice, HDA consists of approximate eqs 2 and 3 with the following ones

4 $$A_{\mathrm{ia},\mathrm{jb}}=\delta_{\mathrm{ij}}\delta_{\mathrm{ab}}\left(\varepsilon_{a}-\varepsilon_{i}-\Delta_{\mathrm{ia}}\right)+\left\langle \mathrm{aj}\right|\mathrm{ib}\rangle +\left\langle a\left|\frac{\partial V_{\mathrm{XC}}}{\partial \rho }j^{*}b\right|i\right\rangle$$

5 $$B_{\mathrm{ia},\mathrm{jb}}=\left\langle \mathrm{ab}\right|\mathrm{ij}\rangle +\left\langle a|\frac{\partial V_{\mathrm{XC}}}{\partial \rho }b^{*}j|i\right\rangle$$

where in expression [Disp-formula eq5], we introduced a diagonal corrective term

6 $$\Delta_{\mathrm{ia}}=\alpha \left\langle \mathrm{ai}\right|\mathrm{ai}\rangle +\alpha \left\langle a\left|\frac{\partial V_{\mathrm{XC}}}{\partial \rho }i^{*}a\right|i\right\rangle$$

So, actually, the hybrid correction is used only for the diagonal elements of matrix *A*, and it can be interpreted as a correction of the difference between the eigenvalues of the virtual-occupied KS orbitals. Although we refer to the original work for a more complete discussion and justification of HDA,^22^ in practice, the main effect of the diagonal correction is to recover the too high energy of the virtual orbital, which is pushed up by the nonlocal Fock exchange.

Because the HDA modifies only orbital energy differences, it can be directly applied to the polTDDFT scheme, where the photoabsorption spectrum σ­(ω) is discretized and computed in a limited number of points from the imaginary part of the dynamic polarizability α­(ω)

7 σ(ω)=4πωcIm[α(ω)]

In the following, we give a brief summary of the polTDDFT algorithm, while we refer the reader to the original literature for more details concerning the method and its implementation.^12–14^ The complex dynamical polarizability is calculated by solving the following nonhomogeneous linear system

8 [S−M(ω)]b=d

In eq 9, **S** is the overlap matrix between density fitting functions, **b** is the unknown vector containing the expansion coefficients *b*_μ_(ω) of the induced density ρ_*z*_^(1)^, **d** is the frequency-dependent vector corresponding to the known nonhomogeneous term, and finally, the elements of the frequency-dependent matrix **M** are

9 $$M_{\mu \nu }=\left\langle f_{\mu }\right|\chi_{\mathrm{KS}}\left(\omega \right)K\left|f_{\nu }\right\rangle$$

In eq 10, χ_KS_ refers to the Kohn–Sham frequency-dependent dielectric function and *K* to the kernel. The original characteristic of the polTDDFT method is the introduction of a simple approximation, which enables the construction of **M**(ω) as a linear combination of frequency-independent matrices **G** ^*k*^ with frequency-dependent coefficients *s*_*k*_(ω), with the following expression

10 $$M\left(\omega \right)=\sum_{k}s_{k}\left(\omega \right)G^{k}$$

With this idea, a set of matrices {**G** ^*k*^} is calculated and stored only once at the beginning, then the matrix **M**(ω) is calculated very rapidly at each photon energy ω, as a linear combination of the {**G** ^*k*^} matrices with the following coefficients as the left-hand side of eq 12

11 $$s_{k}\left(\omega \right)=\frac{4\left(\varepsilon_{a}-\varepsilon_{i}\right)}{\omega^{2}-{\left(\varepsilon_{a}-\varepsilon_{i}\right)}^{2}}\overset{\mathrm{HDA}}{\Rightarrow }s_{k}\left(\omega \right)=\frac{4\left(\varepsilon_{a}-\varepsilon_{i}-\Delta_{\mathrm{ia}}\right)}{\omega^{2}-{\left(\varepsilon_{a}-\varepsilon_{i}-\Delta_{\mathrm{ia}}\right)}^{2}}$$

In order to apply the HDA to the polTDDFT, it is sufficient to correct the orbital energy differences with the same correction term ([Disp-formula eq7]) as already done for expression [Disp-formula eq5]: ε*_a_*– ε*_i_* – Δ*_ia_*, so that the right-hand side of eq 12 is obtained.

Extending HDA to range-separated (RS) functionals requires modifying the Δ*_ia_* term to reflect the RS exchange structure. In order to give an explicit expression for these corrections, it is convenient to recall the split of the Coulomb operator into a Short Range (SR) and a Long Range (LR) component

12 $$\frac{1}{r_{12}}=\mathrm{SR}+\mathrm{LR}=\frac{w\left(\gamma ,r_{12}\right)}{r_{12}}+\frac{1-w\left(\gamma ,r_{12}\right)}{r_{12}}$$

where in ([Disp-formula eq13]) *w* is a switching function which goes to 1 as *r*_12_ goes to zero and goes to 0 as *r*_12_ goes to infinity, then the exchange is calculated at the DFT level for the SR part, and at the exact exchange level for the LR. The standard RS CAM-B3LYP^29^ for GTO basis set employs the complementary error function erfc as *w* switching function; such a choice is essentially technical due to the possibility of integrating analytically. On the other hand, when STO basis functions are employed, as in the present case, it is more convenient to use an exponential switching, which can be integrated analytically for 2-center STO functions; in this case, the CAMY-B3LYP functional is obtained,^27^ where Y refers to the Yukawa potential $\frac{\exp\left(-\gamma r_{12}\right)}{r_{12}}$ .

In practice, we efficiently calculate the correction as follows:

13 $$\Delta_{\mathrm{ia}}=\left\langle \mathrm{ai}\left|\frac{1-\exp\left(-\gamma r_{12}\right)}{r_{12}}\right|\mathrm{ai}\right\rangle -\left\langle a\left|\frac{\partial V_{X}^{Y}}{\partial \rho }\right|i\right\rangle +\left\langle a\left|\frac{\partial V_{\mathrm{XC}}^{\mathrm{ALDA}}}{\partial \rho }\right|i\right\rangle$$

where the first term in eq 14 is the diagonal element calculated with the Long Range attenuated Yukawa potential, the second term is calculated with the Short Range DFT Yukawa functional, and the third term subtracts the ALDA kernel from the diagonal elements in order to avoid double counting. While for the calculation of the second term, an efficient routine is already available in AMS, the first term is calculated with the Resolution of the Identity technique^23^ as follows:

14 $$\left\langle \mathrm{ai}\left|\frac{1-\exp\left(-\gamma r_{12}\right)}{r_{12}}\right|\mathrm{ai}\right\rangle =\iint \frac{\varphi_{a}^{*}\left(\vec{r_{1}\;}\right)\varphi_{a}\left(\vec{r_{1}\;}\right)\left(1-\exp\left(-\gamma r_{12}\right)\right)\varphi_{i}^{*}\left(\vec{r_{2}\;}\right)\varphi_{i}\left(\vec{r_{2}\;}\right)}{r_{12}}d\vec{r_{1}\;}d\vec{r_{2}\;}$$

The numerator in eq 15 contains the product of the electron densities of orbitals φ*_a_* and φ*_i_*

15 $$\rho_{a}\left(\vec{r_{1}\;}\right)=\varphi_{a}^{*}\left(\vec{r_{1}\;}\right)\varphi_{a}\left(\vec{r_{1}\;}\right)$$

16 $$\rho_{i}\left(\vec{r_{2}\;}\right)=\varphi_{i}^{*}\left(\vec{r_{2}\;}\right)\varphi_{i}\left(\vec{r_{2}\;}\right)$$

They are developed as a linear combination of density fitting functions as follows:

17 $$\rho_{a}\left(\vec{r_{1}\;}\right)≅\sum_{k=1}^{N_{F}}f_{k}\left(\vec{r_{1}\;}\right)C_{\mathrm{ka}},\rho_{i}\left(\vec{r_{2}\;}\right)≅\sum_{m=1}^{N_{F}}f_{m}\left(\vec{r_{2}\;}\right)C_{\mathrm{mi}}$$

Using expressions [Disp-formula eq16]–[Disp-formula eq18] to rewrite eq 15, we get

18 $$\left\langle \mathrm{ai}\left|\frac{1-\exp\left(-\gamma r_{12}\right)}{r_{12}}\right|\mathrm{ai}\right\rangle ≅\sum_{k=1}^{N_{F}}\sum_{m=1}^{N_{F}}\iint \frac{f_{k}\left(\vec{r_{1}\;}\right)C_{\mathrm{ka}}\left(1-\exp\left(-\gamma r_{12}\right)\right)f_{m}\left(\vec{r_{2}\;}\right)C_{\mathrm{mi}}}{r_{12}}d\vec{r_{1}\;}d\vec{r_{2}\;}$$

Introducing matrix ***F*** ^***Y***^ with elements

19 $$F_{\mathrm{km}}^{Y}=\iint \frac{f_{k}\left(\vec{r_{1}\;}\right)\left(1-\exp\left(-\gamma r_{12}\right)\right)f_{m}\left(\vec{r_{2}\;}\right)}{r_{12}}d\vec{r_{1}\;}d\vec{r_{2}\;}$$

It is worth noting that such a procedure is possible since an analytical expression for the one- and two-center integrals ([Disp-formula eq20]) is available for STO functions.^27^ After computing ***F*** ^***Y***^, the correct diagonal integrals are finally obtained

19a $$\left\langle \mathrm{ai}\left|\frac{1-\exp\left(-\gamma r_{12}\right)}{r_{12}}\right|\mathrm{ai}\right\rangle ≅\sum_{k=1}^{N_{F}}\sum_{m=1}^{N_{F}}C_{\mathrm{ak}}^{+}F_{\mathrm{km}}^{Y}C_{\mathrm{mi}}$$

The *C* coefficients to be employed in eq 19a are extracted from the projection of the orbital density $\rho_{a}\left(\vec{r_{1}\;}\right)$ on the set of fit functions *f*_*p*_

20 $$\left\langle f_{p}|\rho_{a}\right\rangle =\sum_{k=1}^{N_{F}}\left\langle f_{p}|f_{k}\right\rangle C_{\mathrm{ka}}=\sum_{k=1}^{N_{F}}S_{\mathrm{pk}}^{f}C_{\mathrm{ka}}=d_{\mathrm{pa}}$$

where *S* ^*f*^ is the overlap matrix between fitting function pairs. Solving the linear system ([Disp-formula eq21]) yields coefficients for virtual orbitals; a similar procedure holds for occupied orbitals, finally

21 $$\sum_{n=1}^{N_{F}}{\left(S^{f}\right)}_{\mathrm{kn}}^{-1}d_{\mathrm{na}}=C_{\mathrm{ka}},,\sum_{p=1}^{N_{F}}{\left(S^{f}\right)}_{\mathrm{mp}}^{-1}d_{\mathrm{pi}}=C_{\mathrm{mi}}$$

so that we can rewrite eq 20

22 $$\left\langle \mathrm{ai}\left|\frac{1-\exp\left(-\gamma r_{12}\right)}{r_{12}}\right|\mathrm{ai}\right\rangle ≅\sum_{n=1}^{N_{F}}\sum_{p=1}^{N_{F}}\sum_{k=1}^{N_{F}}\sum_{m=1}^{N_{F}}d_{\mathrm{an}}^{†}{\left(S^{f}\right)}_{\mathrm{nk}}^{-1}F_{\mathrm{km}}^{Y}{\left(S^{f}\right)}_{\mathrm{mp}}^{-1}d_{\mathrm{pi}}$$

Moreover, the effect of the DFT SR kernel can be included since its matrix over the auxiliary density fitting basis is already available, since it is needed by the polTDDFT (see matrix ***Z*** in ref 12)­

23 $$Q={\left(S^{f}\right)}^{-1}\left(F^{Y}+Z\right){\left(S^{f}\right)}^{-1}$$

The final correction for the diagonal term is then obtained as follows:

24 $$≅d_{a}^{+}Qd_{i}$$

For the details of the implementation, we refer the reader to the previous work on the hybrid functionals, since that part is identical, only the matrix ***F*** is substituted by ***F** ^**Y**^*.

An important modification introduced in the present implementation concerns the normalization of the orbital densities, while expressions [Disp-formula eq16] and 17 are normalized; when expanded with auxiliary density fitting basis with expression [Disp-formula eq18], the densities are no more exactly normalized, unless a constraint is introduced in order to impose normalization. Unlike previous versions, we now impose that these densities integrate to unity over $R^{3}$ . This is achieved using a standard Lagrange multiplier approach. The usefulness of the imposition of the normalization constraint to the fitted density has been known for a long time in the context of the efficient calculation of the Hartree potential during the SCF cycles.^30^ In matrix form, it consists of adding an “edge” to the overlap matrix with the normalization integrals of the fitting functions (last column and last row) and putting the value of the normalization constraint (1 in the present case) as the last new element of the known term (which now has dimension N + 1). This new matrix will be no more definite positive, so the Cholesky decomposition cannot be used; thus, the more general standard routine for symmetric linear systems must be chosen instead. We noticed that with this constraint, the agreement between the polTDDFT results and a Casida reference is improved; therefore, starting with the AMS2025 version, this constraint is the default for both hybrid and RS polTDDFT fitted HDA calculations.

## 3 Computational Details

All of the DFT KS calculations reported in this work were performed employing a TZP basis of STO functions (included in the ADF database) and the CAMY-B3LYP hybrid xc-functional.^27^ The auxiliary density fitting basis STO functions are taken from the POLTDDFT basis set database included in the AMS program.^31^ The TDDFT calculations were performed with the polTDDFT algorithm.^12,13^ The HDA density functional approximation^22^ is the only available scheme for hybrid or RS functional within polTDDFT. The HDA has been implemented by exploiting the parallelization at the general Message Passing Interface (MPI) level. The imaginary part of the photon energy was set to 0.075 eV during the calculation of the polTDDFT spectra. All of the calculations were performed with a local developer version of the AMS code, which has already been distributed starting with the AMS2025.01 version. The coordinates of the systems considered in the present work have been taken from the literature.^32,33^

## 4 Results and Discussion

The aim of the present work is the implementation of the RS functional within TDDFT, in the framework of the polTDDFT algorithm using the HDA approximation. Accordingly, the new method must be validated in two main directions. First, we assess whether the algorithm is capable of accurately describing charge-transfer (CT) excitations. Second, we evaluate its computational performance and scalability to ensure it can be applied to systems where conventional approaches are computationally prohibitive.

To address the first point, we consider in Section 4.1 the ethylene–tetrafluoroethylene adduct, which is a typical model to test CT excitation. The second point is discussed in Section 4.2, where we consider an extended system (TAA-th-BTD-th-AQ) comprising one donor and two acceptor fragments so that the CT excitations consist of intramolecular transitions between spatially separated orbitals.

### 4.1 Ethylene–Tetrafluoroethylene

The ethylene–tetrafluoroethylene adduct is a practical model used by many authors^21,32,34,35^ to test the suitability of theoretical methods to describe CT excitations. In this work, our goal is to test the accuracy of the present implementation, so we will take the Casida results as a reference and will compare them with the new polTDDFT implementation, using the same basis set, exchange–correlation functional, and code (AMS) in both calculations. In this work, the distance between the two fragments has been set to 5 Å. It is convenient to start describing the Casida results, which are reported in Table 1 and Figure 1, while the orbitals involved in the excitations are reported in Figure 2. Notice that the polTDDFT method directly gives a continuous profile, while the Casida one gives excitation energies and oscillator strengths, which are reported in the figure as vertical bars. In order to facilitate the comparison between the Casida method and the polTDDFT, we have also reported in the figure the profile obtained by smoothing the Casida discrete excitations with Lorentzian functions with the same HWHM as the imaginary photon energy used in polTDDFT in order to have the same bandwidth in both methods. From Table 1 and Figure 1, it is worth noting that the most intense transitions, at 7.55 eV (*f* = 0.22) and 8.32 eV (*f* = 0.38), correspond to intramolecular π → π* on ethylene and tetrafluoroethylene, respectively. The first excitation at 6.64 eV, which is rather weak (*f* = 0.024) but still visible on the scale of Figure 2, is an intramolecular transition π → Rydberg F 3s on tetrafluoroethylene. It is worth noting that RS functionals, besides giving a correct description of the CT excitations, also display the correct behavior of the exchange at large distances and therefore properly support the Rydberg states. Moreover, such Rydberg states to be supported need that the basis set contains diffuse functions, as in the case of the presently employed TZP. The next visible excitation at 7.10 eV consists of a CT from the π orbital of ethylene to the Rydberg F 3s orbital of tetrafluoroethylene; the relatively high intensity (*f* = 0.11) is a consequence of the very diffuse Rydberg state. In fact, in general, CT transitions involving valence orbitals are rather weak, since the initial and final orbitals are localized in different spatial regions, so their electric dipole transition moment tends to zero as their separation increases. On the other hand, Rydberg orbitals are very diffuse, so they can be more effective than valence orbitals to overlap with moieties that are localized far away. In fact, as we will see later, the other CT that involves valence instead of Rydberg states is less intense by at least a factor of 2. The next transition at 7.48 eV is again a CT, but this time it is from tetrafluoroethylene π → ethylene π*, with intensity *f* = 0.033, roughly three times weaker than the previous Rydberg CT. The following CT falls at 8.39 (*f* = 0.015) and corresponds to ethylene π → tetrafluoroethylene π*; however, such a transition is very close in energy to the strong intramolecular one at 8.32 eV, which is 25 times more intense. In Figure 1, the comparison between the Casida and present polTDDFT implementation is presented as well, while both excitation energies and oscillator strengths are reported in Table 1 for a more quantitative comparison between the two approaches. The polTDDFT spectrum satisfactorily reproduces the main features of the Casida spectrum, although some discrepancies are present, in terms of energy shifts as well as intensity redistribution. Starting with the low energy region, the two excitations calculated by Casida at 6.64 and 7.10 eV are found at 6.68 and 7.12 eV, respectively, in the polTDDFT calculation, so we obtain an excellent energy match but a clear deterioration of the intensity, whose distribution is different. Actually, the polTDDFT profile gives a feature at 6.96 eV, which is not apparent in the Casida spectrum; however, from Table 1, a dark transition (with negligible oscillator strength) is found at 6.76 eV. So, we can rationalize the presence of this feature due to an incorrect redistribution of the oscillator strength between the two states: in Casida, one excited state brings all of the intensity, while in polTDDFT, the intensity is shared. This effect is typical when the excited states are very close to each other in energy; in fact, going to the limit of two degenerate states, it is possible to choose a unitary transformation that preserves their eigenvector nature but brings the intensity from one to the other. If the states are not exactly degenerate, a small perturbation/inaccuracy may strongly mix the states, changing the intensity distribution between the two states. Of course, this difficulty ascribed to polTDDFT is more critical for small systems, when the states are few and well separated in energy; however, when the system is larger, the density of excited states increases, they start to be almost degenerate, and polTDDFT will furnish an average description, even more practical than the Casida approach when the presence of many congested excited states prevents a simple analysis. Since polTDDFT has been designed suitably for applications to very large systems containing several hundreds of atoms, we believe that this does not represent a real limitation of the method. The next features at the Casida level fall at 7.48 and 7.55 eV, in polTDDFT, we assign them with the reverse order at 7.66 and 7.46 eV. Such an assignment is corroborated by 3 different arguments: first, the same dominant one-electron excited configuration, second, the intensity (which is also reversed), and third, by the following fragment analysis. In fact, with polTDDFT, it is possible to perform a fragment analysis splitting^36^ of the spectrum into 4 components, according to the initial and final fragment. In the present situation, it is quite natural to split the system into two fragments corresponding to ethylene and tetrafluoroethylene in order to identify the CT region. In Figure 3, we report the total fragment analysis, and also the partial analysis according to the *x* or *z* active electric dipole component, since the *y* component is not active for these transitions. The CT regions in Figure 3 are filled in yellow and green for ethylene → tetrafluoroethylene and tetrafluoroethylene → ethylene, respectively; this corroborates attributing the polTDDFT peak at 7.66 eV to the CT Casida peak at 7.48 eV. Interestingly, the ethylene → tetrafluoroethylene CT shows up around 8.4 eV in the *x* dipole component and 7.2 eV in the *z* component: the former corresponds to the 8.39 eV peak in Casida (HOMO – 1 π → LUMO + 2 π*) and the latter to the 7.1 eV peak in Casida (HOMO – 1 π → LUMO + 1 Ryd). Notice that while the π → π* transitions are active with the *x* component, the π → F 3s Rydberg are active in the *z* dipole component due to the totally symmetric shape of the Rydberg orbital. Finally, the Casida peaks at 8.0 and 8.32 eV are shifted at higher energy by 0.2 eV in the polTDDFT result, but keeping the correct intensity distribution.

**Figure 1:**
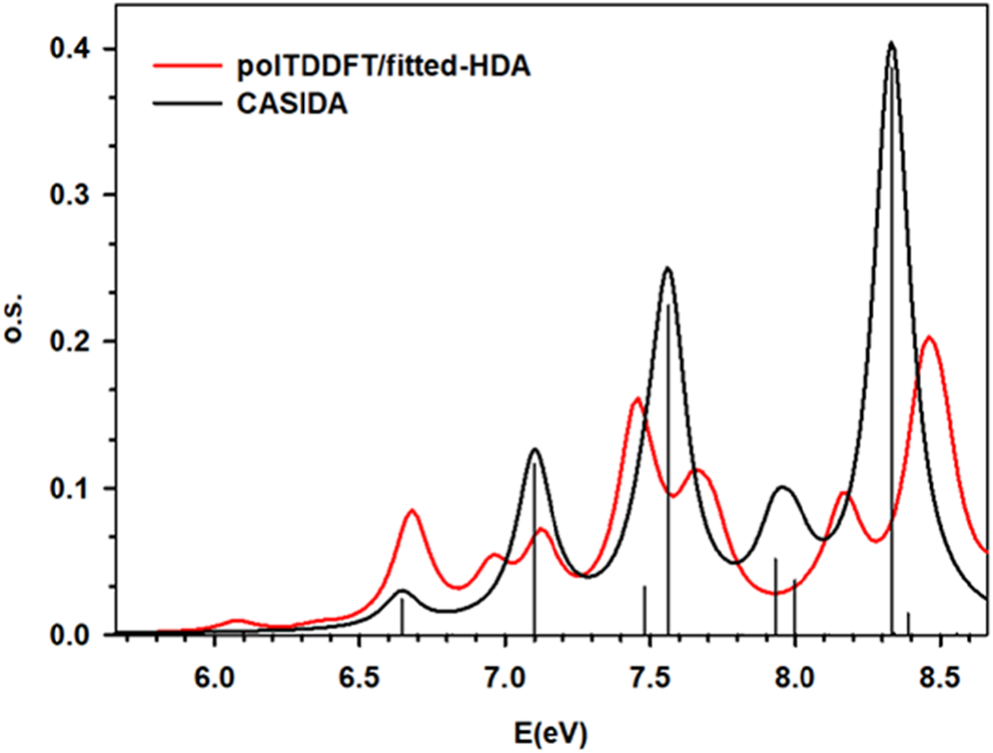
Calculated photoabsorption spectrum of the ethylene–tetrafluoroethylene model system at a distance of 5Å, absolute intensity (oscillator strength) on the vertical axis, excitation energy on the horizontal axis. Black vertical bar: discrete Casida excitations, black curve: smoothed Casida spectrum, red curve: polTDDFT.

**Figure 2:**
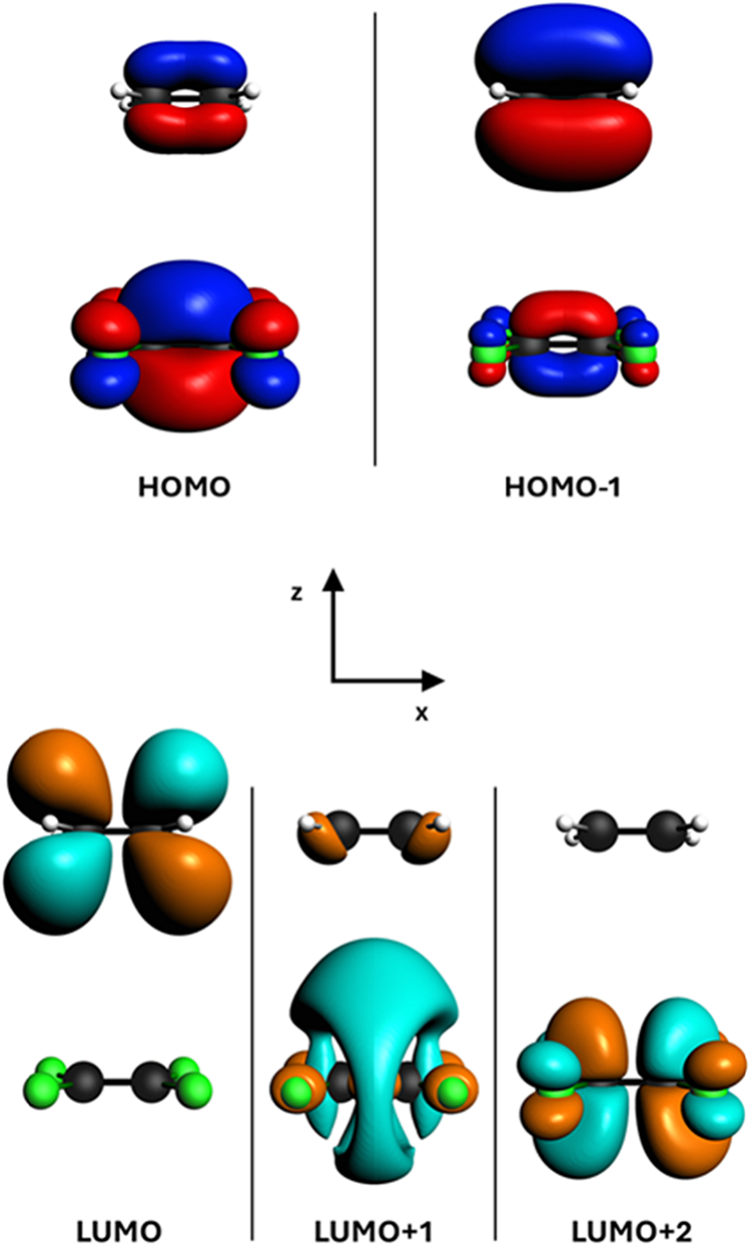
Occupied and virtual orbitals of the ethylene–tetrafluoroethylene model system involved in the most important excitations.

**Figure 3:**
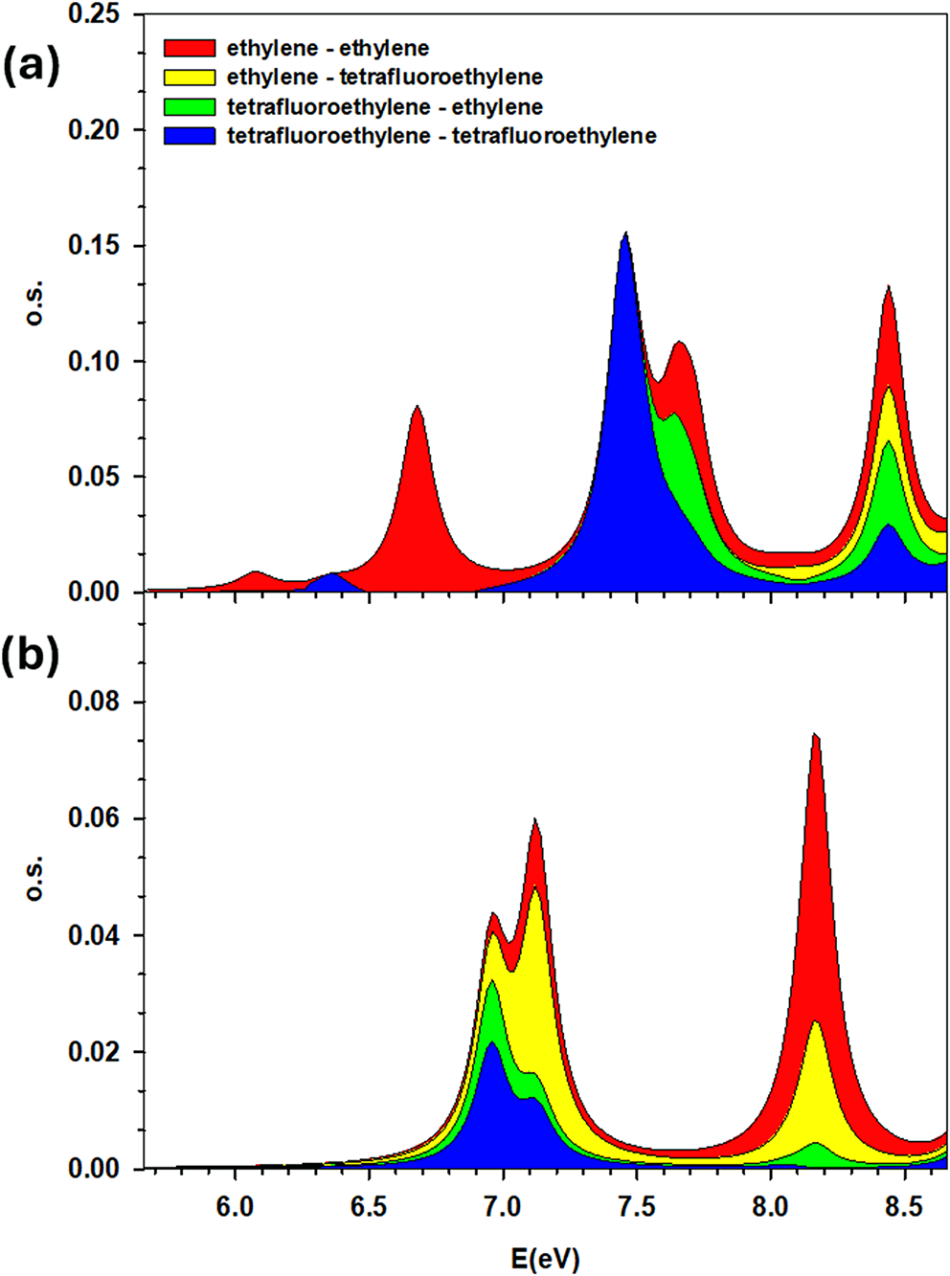
Fragment analysis of the spectrum of the ethylene–tetrafluoroethylene model system. (a) Upper panel: x component of transition moment electric dipole, (b) lower panel: z component of transition moment electric dipole.

**Table 1:**
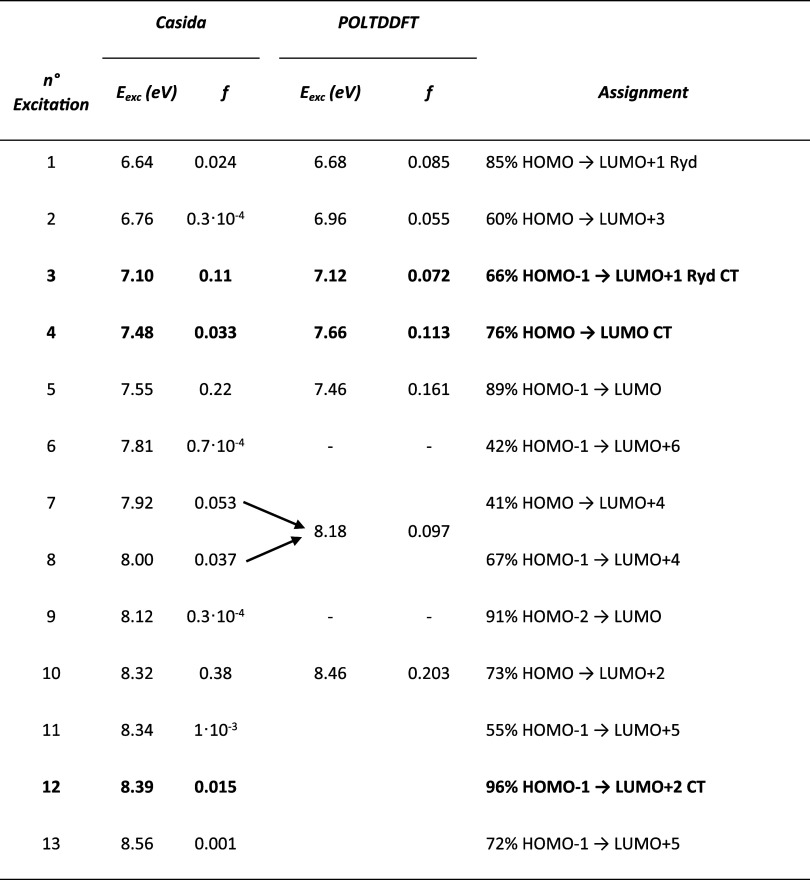
Lowest Excitation Energies (*E*_exc_) and Intensities in Terms of Oscillator Strength (*f*) of the Ethylene–Tetrafluoroethylene Adduct at a Distance of 5Å Calculated with Both Casida and polTDDFT Methods^a^

a Assignment in terms of the dominant one-electron excited configuration. Calculated at the TDDFT level with CAMY-B3LYP XC-functional and TZP STO basis set. CT excitations are in bold.

We can conclude the analysis on this model system, saying that the present implementation of the polTDDFT RS CAMY-B3LYP functional is able to describe the CT transition, although some minor deterioration with respect to the Casida reference is found. The discrepancy in the energy is rather small, no more than 0.2 eV at most; more sensitive is the intensity, which is usually well reproduced, but in some instances (low energy part of the spectrum), more evident deviations are observed. Such still minor deterioration can be safely attributed to deficiencies of the density fitting basis set, which is optimized to reproduce the spectrum but has not yet been optimized to fit the densities as in previous eq 18. An analogous deterioration for the same problem has been observed for the fitted HDA with global hybrid functionals,^23^ but such deficiency tends to strongly alleviate when the size of the system increases.^25^ The systematic improvement of the accuracy as the size (or the number of electrons) increases has also been observed in different simplified TDDFT schemes, in particular the recent XsTD-DFT from de Wergifosse and Grimme.^37^ In particular, for the XsTD-DFT scheme, a complete analysis on a set of 77 molecules and 4 different functionals has demonstrated that the excitation energy absolute deviations are decreasing as the number of electrons increases. The effect is quite prominent, starting with errors on the order of 1–2 eV for systems with less than 50 electrons, going down to 0.2 eV when 250 electrons are present. However, in the paper,^37^ this interesting trend was identified but not explained. We can tentatively explain this behavior as follows: assume that the orbitals are all strongly delocalized; this means that in larger systems, the orbitals are in any case more flexible since they are contributed by more basis set functions. Since the present accuracy is governed by the accuracy of the two-electron integrals, the latter will be more accurate when the system is larger. Actually, this is a very interesting finding that deserves a deeper analysis in future work; in fact, if well understood, this may allow us to improve even more both accuracy and efficiency for very large systems.

So we are confident that the same improvement with system size should be expected for RS functionals as well, preventing the necessity to reoptimize the density fitting basis set when larger systems are taken into account. Although the polTDDFT method is much faster than the Casida one, for the present model system, the computational effort is negligible for both approaches, employing only a few seconds to complete the calculations; therefore, we defer the analysis of the computational economy to the next system, which is much larger and therefore more informative.

### 4.2 Electron-Donor–Acceptor–Acceptor Triad

In order to test the present implementation on a challenging system, we have selected a so-called electron-donor–acceptor–acceptor triad.^33^ Such a system is built (see Figure 4) by three different fragments linked by a thiophene moiety: the first fragment is a Donor (D) and consists of a triarylamine (TAA), linked by thiophene (th) to the first Acceptor (A1) consisting of a benzothiadiazole (BTD) further linked by th to the second Acceptor (A2) consisting of an anthraquinone (AQ). Such a complex system is very interesting as a model of a molecular electronic circuit. The current is triggered by a bright CT transition by light absorption from D TAA to A1 BTD, followed by a nonradiative electron transfer from A1 BTD to A2 AQ. The so-obtained charge-separated system (hole in TAA and electron in AQ) promotes a strong geometrical relaxation such that D and A2 get much closer. Finally, the hole–electron recombination between D and A2 takes place, and the electron returns in its initial state, closing this sort of molecular electronic circuit. Since our goal is to test the CT performance of this method, we focus only on the first step of the process, namely, the photoabsorption spectrum, which we compare with the experimental one to test if our approach is able to properly describe the CT excitation from D to A1. The experimental photoabsorption spectrum^33^ is compared with present polTDDFT calculations in Figure 5, while the analysis of the most relevant calculated spectral features is reported in Table 2 at both Casida as well as polTDDFT levels. Such analysis is performed in terms of leading one-electron excited configurations, so the molecular orbitals involved are reported in Figure 6. In the following, we will employ the polTDDFT to discuss the results. The lowest energy calculated peak is found at 3.16 eV, which is essentially contributed by HOMO → LUMO + 1, from Figure 6. Such a transition can be easily assigned to a CT excitation from D (TAA) to A1 (BTD). In the experiment,^33^ such peaks are found at 415 nm, corresponding to 3.02 eV, in excellent agreement with the present calculation. Such a nice result confirms the suitability and the accuracy of the present scheme to describe CT excitation in large systems. The next transition is calculated at 3.42 eV and corresponds to the HOMO – 2 → LUMO excitation: from Figure 6, it can be ascribed to an A1 (BTD) to A2 (AQ) transition, but with strong involvement of the th linker, so with only minor CT character; in fact, its intensity is much larger than the first excitation. The experimental peak falls at 355 nm, corresponding to 3.49 eV; also, in this case, theory is in fairly nice agreement with the experiment. The next peak calculated at 3.74 eV displays a strongly mixed character (Table 2), which can be ascribed on average to a BTD-th → BTD transition. We do not have a clear indication in the experiment for such a peak; however, the previous feature at 3.49 eV is rather flat at the maximum, so it is possible that these two transitions are both encompassed by the feature around 3.49 eV. Actually, in the assignment given in the experimental work, the feature at 3.49 is ascribed to a th → BTD transition, which is consistent with the present work.

**Figure 4:**
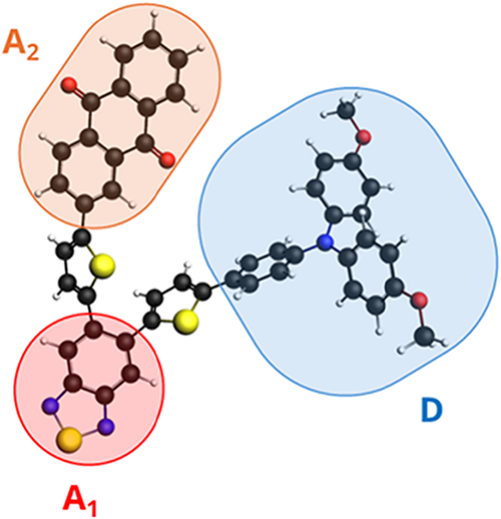
Geometry of the TAA-th-BTD-th-AQ electron-donor–acceptor–acceptor triad.

**Figure 5:**
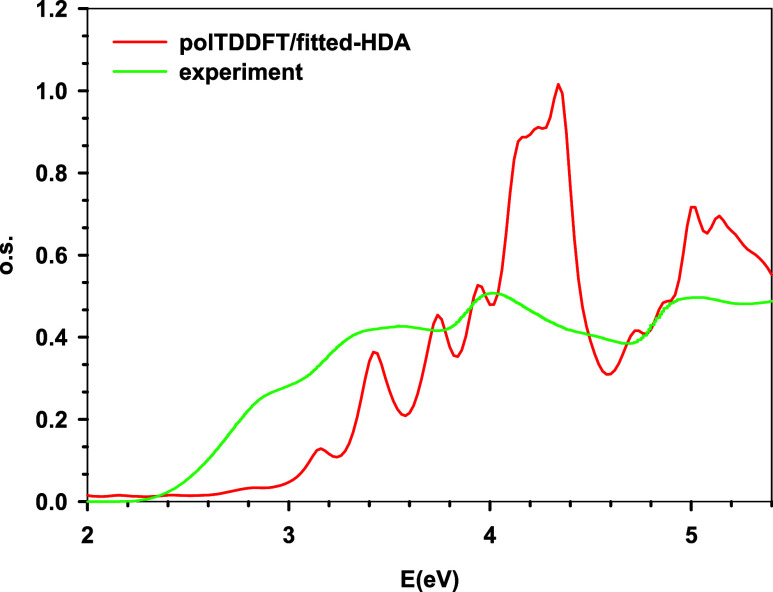
Calculated photoabsorption spectrum of the TAA-th-BTD-th-AQ electron-donor–acceptor–acceptor triad, absolute intensity (oscillator strength) on the vertical axis, excitation energy on the horizontal axis. Red curve: polTDDFT, green curve: experiment from ref 33.

**Figure 6:**
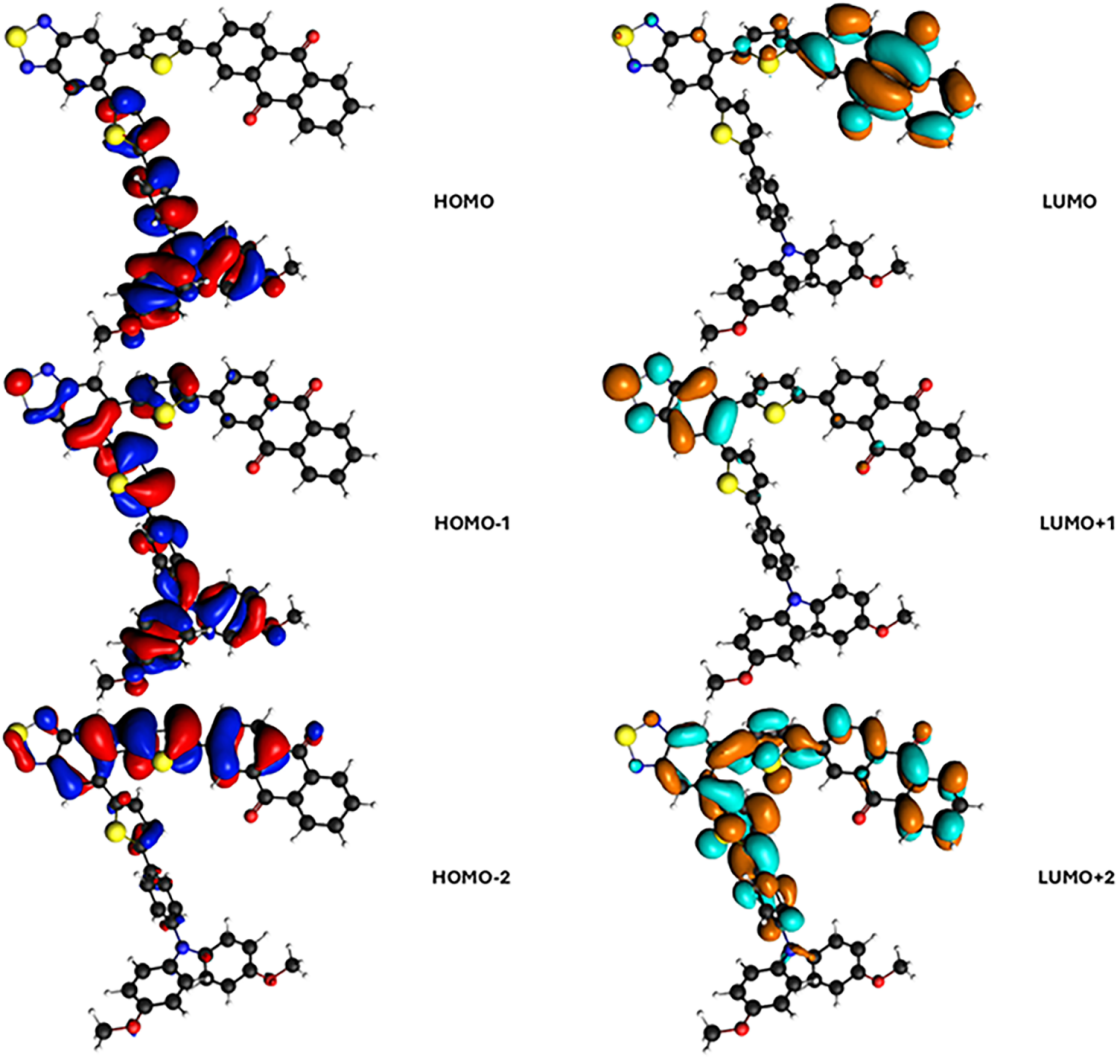
Occupied and virtual orbitals of the TAA-th-BTD-th-AQ electron-donor–acceptor–acceptor triad involved in the most important excitations.

**Table 2:** Main Absorption Peaks Predicted by Both Casida and polTDDFT/fitted-HDA for TAA-th_BTD-th-AQ System^a^

	casida		POLTDDFT		*a*
absorption peak	*E*_exc_ (eV)	*f*	*E*_exc_ (eV)	*f*	assignment
A	2.98	0.27	3.16	0.13	72.44% HOMO → LUMO + 1
B	3.32	0.31	3.42	0.36	66.27% HOMO – 2 → LUMO
C	3.73	0.32	3.74	0.45	34.76% HOMO – 1 → LUMO + 1
					27.81% HOMO – 2 → LUMO + 1
D	3.54	0.49	3.94	0.53	83.28% HOMO → LUMO + 3
E	4.24	0.48	4.16	0.89	51.30% HOMO → LUMO + 4
F	4.38	0.25	4.24	0.91	37.67% HOMO → LUMO + 9
					14.57% HOMO → LUMO + 4
G	4.48	0.18	4.34	1.02	47.82% HOMO – 2 → LUMO + 2
					21.03% HOMO – 15 → LUMO
H			4.72	0.42	40.89% HOMO – 1 → LUMO + 3
					13.07% HOMO – 1 → LUMO + 2
I			4.88	0.49	49.75% HOMO – 2 → LUMO + 3
					15.39% HOMO – 8 → LUMO + 1
L			5.00	0.72	51.37% HOMO – 1 → LUMO + 4
M			5.14	0.70	39.42% HOMO – 1 → LUMO + 4
					11.61% HOMO – 2 → LUMO + 3

a For each one, we report the excitation energy, the oscillator strength, and the assignment. Assignment in terms of the dominant one-electron excited configuration.

Going to higher energies, we find four features very close to each other in energy, from 3.95 to 4.34 eV, which we can compare with the experimental maximum at 4.02 eV. All of these transitions display a rather high contribution of th in both initial and final orbitals. Then, a rather wide energy gap is found in both calculated and experimental spectra. The next features are found in the calculated spectrum from 4.72 to 5.14 eV, with the most intense being at 5.00 eV; in the experiment, the maximum is found at 5.04 eV, confirming the accuracy of the present theoretical model. For such a region, the inspection of the molecular orbitals involved suggests that the transitions are internal to acceptor fragments, namely, A1 (BTD → BTD) and A2 (AQ → AQ). This explains the higher intensity for this energy region. We can supplement this section with the fragment analysis reported in Figure 7. In the upper figure, we divided the molecule into 2 fragments: the first fragment corresponds to the TAA donor, while the second fragment includes all of the remaining moieties: BTD, both bridges, and AQ. It is well evident that the first peak corresponds to a CT from TAA (as previously mentioned). In order to specify better the involvement of the fragments, in the lower panel we split the molecule into 5 fragments (accordingly to their chemical nature: D, A1, A2, and the two th bridges), but we reported only the contributions from the donor D to any of the two acceptors A1 or A2 and from A1 to A2. From such analysis, the first peak is clearly ascribed to the TAA to BTD CT transfer, as we already did on the grounds of the localization of the molecular orbitals, corroborating previous discussion. We can conclude this section with a comparison between the Casida and the polTDDFT methods for such a larger system, in terms of accuracy as well as computational economy. From Table 2, it is well apparent that the agreement between Casida and polTDDFT is fairly nice in terms of excitation energies; usually, the disagreement is much less than 0.2 eV, with the only exception of the peak D, which is shifted by 0.4 eV. The intensity distribution is more difficult; a rather good agreement is obtained up to 4 eV, but above, the polTDDFT seems to overestimate the intensity. Actually, this is not really true because for such a system, the Casida method furnishes a very high density of states above 4 eV. While polTDDFT provides an average, the Casida gives several almost degenerate discrete transitions that should be properly summed up for a correct comparison with polTDDFT. For such reason, we do not report in Table 2 the Casida states above 4.48 eV. Regarding the computational economy, we ran the calculations up to 6 eV of excitation energy, using 20 cores on a single node of a cluster with an Intel Xeon 20-Core 4416 + 2.0Ghz CPU. The Casida TDDFT took 12 h and 25 min, while polTDDFT took 1 h and 22 min, so polTDDFT proved 9 times faster than Casida. Such a factor is larger as the system becomes larger and larger, so polTDDFT will be faster than Casida by more than 1 order of magnitude. Notice that for very large systems, like for example metal clusters, like Au_25_ protected by ligands, Casida calculations with global hybrid or RS functionals are not practicable at all if a spectrum up to 5 eV of excitation energy is requested; on the other hand, the polTDDFT with HDA approximation and RI technique can deal with such systems with modest efforts (2 days using 20 CPU on the same clusters).

**Figure 7:**
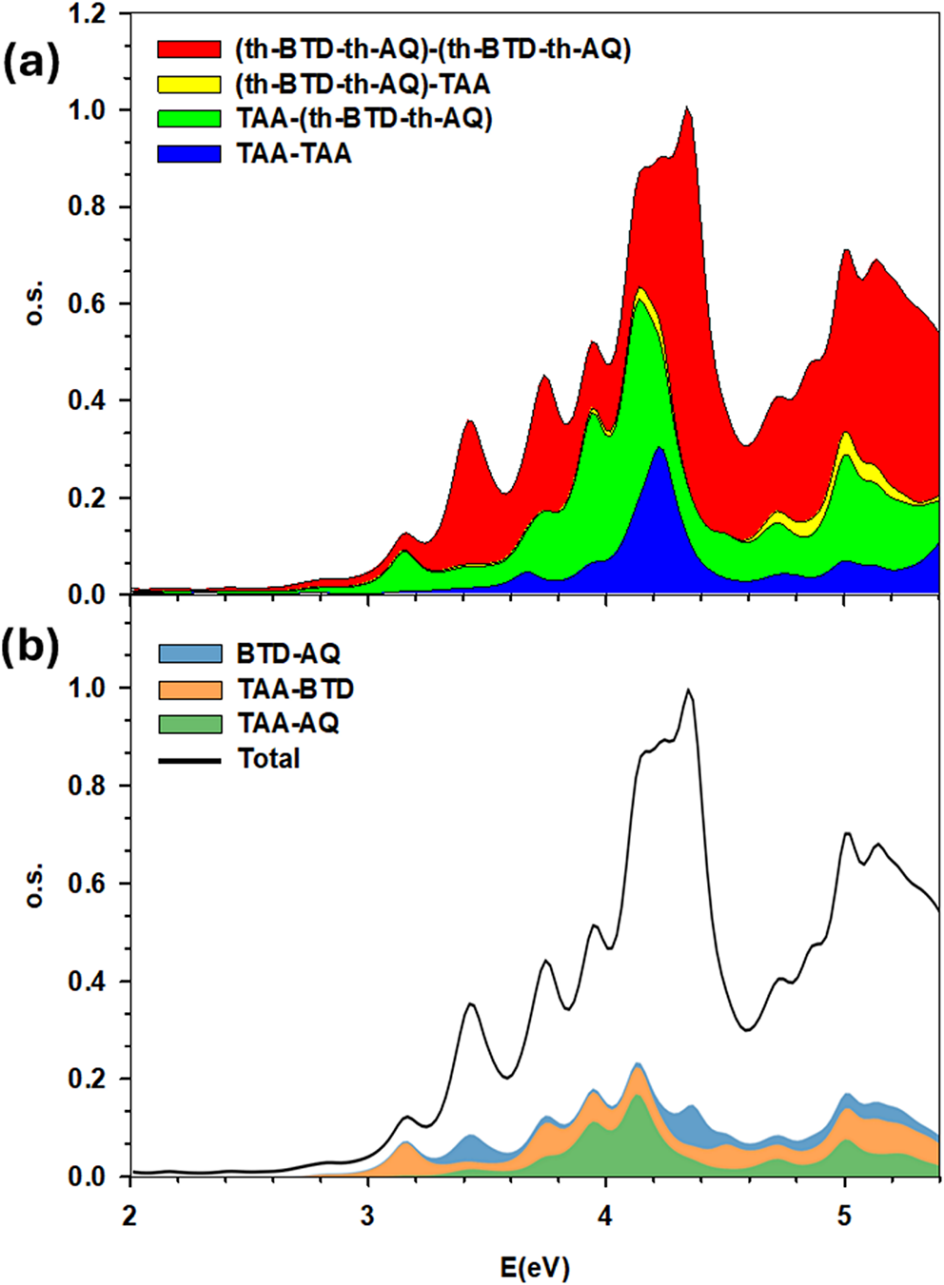
Fragment analysis of the spectrum of TAA-th-BTD-th-AQ electron-donor–acceptor–acceptor triad. In panel (a) the thiophene moiety is included in the fragments, while in panel (b) thiophene is not included.

Finally, we can conclude that the present implementation of the RS CAMY-B3LYP functional in polTDDFT has proven accurate and efficient also for the description of such a complex system, with an accuracy even better than in the previous section model system. The better performance on a larger system in terms of accuracy should be ascribed to the density fitting basis set: as the system under study increases its size, the deficiencies of the basis set become less and less important.

## 5 Conclusions

In this work, we implemented an RI technique to employ RS xc-functionals within the HDA approximation at the TDDFT theory level, solving the equations through the polTDDFT algorithm. Only the diagonal matrix elements of the RS kernel are computed, making the overall computational cost comparable to that of a DFT SCF single-point calculation with hybrid functionals. The method reliably describes CT excitations, as demonstrated for the ethylene/tetrafluoroethylene model system. Moreover, the comparison with respect to the experimental photoabsorption spectra of an electron-donor–acceptor–acceptor triad demonstrates the method’s predictive accuracy and efficiency.

This result suggests that polTDDFT with the RI technique and global or RS hybrid xc-functionals represents a computationally efficient yet accurate approach, and it stands out as a promising tool for investigating large molecular systems, potentially comprising several hundred atoms, with a favorable trade-off between cost and precision.

## Data Availability

The data that support the findings of this study are available from the corresponding author upon reasonable request.
